# Using Qualitative Questionnaires in Medical Education Research

**DOI:** 10.5334/pme.1102

**Published:** 2024-05-06

**Authors:** Michal Tombs, Heather Strange

**Affiliations:** 1 C4ME, School of Medicine, Cardiff University, Neuadd Meirionnydd, Heath Park, Cardiff, UK; 2 Centre for Trials Research, School of Medicine, Cardiff University, Neuadd Meirionnydd, Heath Park, Cardiff, UK

## Abstract

Most students in Health Profession Education courses are new to the world of qualitative research. Faced with the challenge of designing a research project, they are often drawn towards using the questionnaire as a data collection method, commonly assuming that utilising open-ended questions alone constitutes qualitative research design. Designing questionnaires that meet the standards of rigour is challenging, and this common assumption reflects inexperience with and misunderstandings of qualitative ontology, as well as the lack of methodological literature on designing and developing qualitative questionnaires. This paper is written with research supervisors as well as students in mind, as it is aimed to help elucidate the decision-making process and the justification for using a qualitative questionnaire. Drawing upon examples of research conducted by our students, and the wider literature, we demonstrate how qualitative questionnaires can produce rich and meaningful findings when they (1) prioritise qualitative research values, and (2) follow a rigorous design process when the questionnaire is developed. We conclude by offering a simple framework for developing rigorous qualitative questionnaires to those who may consider using this approach.

## Introduction

Questionnaires are a popular data collection method within the field of Health Profession Education (HPE) research and are typically used to collect descriptive data [1]. In comparison, questionnaires that centre on qualitative ontology have often been excluded from discussions within HPE research [2]. This is not surprising as the self-reporting nature of the questionnaire approach is often considered to be at odds with the ontological underpinnings of qualitative enquiry: for the qualitative researcher, data should be placed in context, hold personal meaning, be emotionally and socially nuanced, and layered with detail [3]. The questionnaire method – unlike more discursive, collaborative qualitative data-generation techniques such as interviews or focus groups – does not involve direct interaction with the researcher, and instead asks participants to self-administer and ‘submit’ responses independently. For the pure constructionist researcher, the absence of interaction thus contradicts the iterative epistemological approach of qualitative research.

For students who are also busy clinicians and thus manage many conflicting priorities during their studies, the use of qualitative questionnaires in their student research projects may be appealing due to the multiple practical advantages they offer. However, the justification for the data collection methodology should be guided primarily by the subject of inquiry [3], rather than any practical concerns. To ensure that the data produced (brief written accounts) will be a valuable and appropriate source for examining the phenomena at hand, and multiple perspectives on it, it is important to understand when and how the qualitative questionnaire is best used. Qualitative questionnaires can generate rich contextual data if the researcher is able to harness and prioritise qualitative research values and paradigms throughout the research process [4] and the ontological position taken intends to explore “how people interpret their experiences, how they construct their worlds, and what meaning they attribute to their experiences” [3]. Reflexivity must be attended to, and the development and use of the questionnaire should “meaningfully interconnect literature, research questions/foci, findings, and interpretations with each other.” [5].

Understanding and addressing issues of qualitative research rigour is likely to help prevent novice researchers from assuming that simply inviting participants to answer free-text questions constitutes qualitative research [4] – a misconception that may be attributed partly to the lack of methodologically-focused literature on qualitative questionnaires [2]. We attempt to address the gap in the current methodological literature by providing a summary of when and why qualitative questionnaires may be used in HPE research and offer a simple framework for guiding the design of methodologically sound qualitative questionnaires.

## When and why researchers may choose to use a qualitative questionnaire

Multiple factors influence decisions on whether qualitative questionnaires should be used for a particular project or research question, and it is important to understand potential strengths and limitations before committing to this approach. Here, we consider practical requirements, and the advantages qualitative questionnaires may offer both researchers and participants, highlighting how their anonymous nature may encourage participation and help capture diverse perspectives.

### Practical advantages for researchers and participants

Questionnaires are typically perceived to be a less complex data collection technique when compared to interviews, focus groups or ethnographic observations, placing considerably less pressure on resources and time [6]. If used online, data are collected and recorded using specialist applications and can be easily exported into spreadsheets, and subsequently into qualitative data analysis software, for analysis. Bhatt [7], new to HPE research at the time of writing, provides a useful reflection on such practical advantages in relation to their study on medical students’ perceptions of role model behaviour and their perceived impact. Attempting to capture the diverse voices of medical students in the later stage of their studies, Bhatt used a ‘snowball sampling’ approach to recruit participants, distributing the online survey via links and a QR code. Participants completed the questionnaire via Google Forms using an internet-enabled device of their choice at a time and place convenient for them. When using online questionnaires, the participant consent process is relatively straightforward; in Bhatt’s study, participants were not able to access and complete the questionnaire unless they ticked a box confirming their consent. Once completed, participants submitted responses via a submission link. Email reminders were sent at various time points to try and gain as much data as possible, as it was anticipated that some responses would not be usable due to lack of detail.

The standardized nature of questionnaire design is also considered advantageous for the novice qualitative researcher [6]. Participants are asked the same questions, in the same way and order, and the data generated facilitates comparability and ease of analysis. Google Forms provided Bhatt with automatic data summaries; these were subsequently copied and pasted into an excel sheet to aid comparability of entries, and to examine patterns and themes. Here, the researcher did not need to transcribe audio or video data, and faced no issues of legibility as may be generated by handwritten responses. Text was highlighted, comments were made, and thematic analyses were conducted using the spreadsheet. The accounts may not have been as layered or complex as those typically obtained through other forms of qualitative data collection (e.g. interviews or focus groups), but the data generated still prioritised the voice of the participants and enabled them to elaborate on their experiences and perspectives, attaching personal meaning to the concepts explored. Bhatt was surprised by the rich and meaningful data gathered from the 141 medical students that took part; analysis and interpretation of the questionnaire data categorised educators’ role model behaviours into three themes (‘student rapport’, ‘credible and highly skilled’, ‘humility) and demonstrated how student participants considered these behaviours had significant impact on their learning and professional development, including character modelling, enriched learning, motivation, and career planning.

### Encouraging participation and anonymity

For qualitative researchers, the task of maintaining participant anonymity presents unique challenges [8]. This may be an important factor when deciding to use a qualitative questionnaire, particularly so where the researcher-participant relationship is at risk of power imbalance, which in turn presents a risk to ethical research conduct and the credibility of research findings. In our experience, this is often the case when students enrolled in postgraduate programs conduct research in settings and with people familiar to them. Being an ‘insider’ may be a strength to be leveraged through reflexivity [9], however, power imbalance is a significant factor that can impact credibility [10]. For example, in his initial research design, Taylor [11] proposed to evaluate clinical placements provided at his place of work (an Accident & Emergency department) by interviewing undergraduate medical students who completed the placement. He was transparent when presenting his research proposal to the ethics committee, highlighting potential risks to credibility of findings given his senior roles at both the A&E department and the medical school. In response, the ethics committee highlighted not only the potential risk to data credibility (i.e., participants may not provide a full account of experiences due to the researchers’ seniority and established professional relationships), but also the strong potential for generating psychologically and emotionally sensitive data. As such, the ethics committee did not approve this method of data collection and highlighted the need for an alternative data collection strategy that offered greater anonymity. Using a proxy interviewer was considered, but as this research was conducted for a postgraduate dissertation and required the student to be involved in every aspect of the project, a qualitative questionnaire was proposed and developed. The justification for choosing the qualitative questionnaire is also based on the subject of enquiry [12], and whilst evaluation of clinical placements may not be considered a particularly sensitive topic, it is important to understand that the open nature of qualitative enquiry may indicate otherwise. Even a simple question such as ‘*tell us about your clinical placement at hospital x*’ can evoke a strong emotional reaction if the experience was characterised by extreme, unexpected, or otherwise difficult encounters [13].

The standardisation process utilised by Taylor in developing his qualitative questionnaire helped alleviate the potential risk his dual role as researcher and clinician imposed, and provided a solution for encouraging full, open participation and for maintaining participant anonymity [11]. Using Microsoft Forms, Taylor collected responses from 38 undergraduate students who completed the clinical placement. The invitation was sent via an administrator and no identifiable data were collected to assure anonymity. Students may not have felt safe sharing such experiences directly with someone in a position of power, and the use of an anonymous questionnaire may have alleviated participant anxieties and enabled perspectives to be fully and openly expressed. The resulting data were rich and detailed and enabled Taylor to explore some negative experiences during the placement and their impact on students. Taylor found that interactions with supervisors and peers could be experienced as triggers of shame amongst medical students, with specific examples including derogatory comments, lack of belonging, and being made to feel ‘like a burden’.

### Capturing diverse perspectives

The qualitative researcher prioritizes representation of accounts with depth and complexity rather than being ‘representative’ in the positivist sense [14]; depth and complexity cannot however be guaranteed, particularly when using the qualitative questionnaire. The method is therefore more suitable for studies where the research aim is relatively broad, where data are anticipated to be ‘shallow’, and where dialogue is likely to be weak [2]. Moreover, there is a strong chance that participants may simply write only a few words or provide sentences that lack context and richness. Data may need to be discarded due to lack of specificity [13], thus this method is more suitable for studies where the population of interest is large and diverse [12].

Patrick et al. [15] provides an example of research where the qualitative questionnaire was a good ‘fit’ for the research question, topic, and population. The aim of the study was broad and aimed at testing a theory, in particular the extent to which instructor behaviours were associated with pre-defined theoretical dimensions of leadership. It was also anticipated that participants would be reluctant to engage in dialogue, and that data would be ‘shallow’ due to the hierarchical nature of the research environment, and sensitivities of roles (military recruits and their instructors).

Requiring minimal administrative oversight, qualitative questionnaires can be distributed widely and hold the potential to reach under-represented and diverse populations, many of whom may not choose to participate in interviews or focus groups due to practical, cultural, or psycho-social considerations [10]. Attempting to capture the voices of participants at different levels of training, spread across many geographical locations across the UK from all three-armed forces (i.e., Army, Royal Navy and Royal Airforce), Patrick et al. collected survey responses from 1,495 participants. As expected, not all accounts were detailed or specific enough, and 345 responses were disregarded. The final sample of 1,150 generated rich data from instructors and trainees. Even though participants provided relatively brief answers, the open-ended format enabled them to provide meaningful accounts, in their own language, thus allowing space for their perspectives and explanatory frameworks to be expressed and prioritized [6].

## Developing methodologically sound qualitative questionnaires

**Figure 1:**
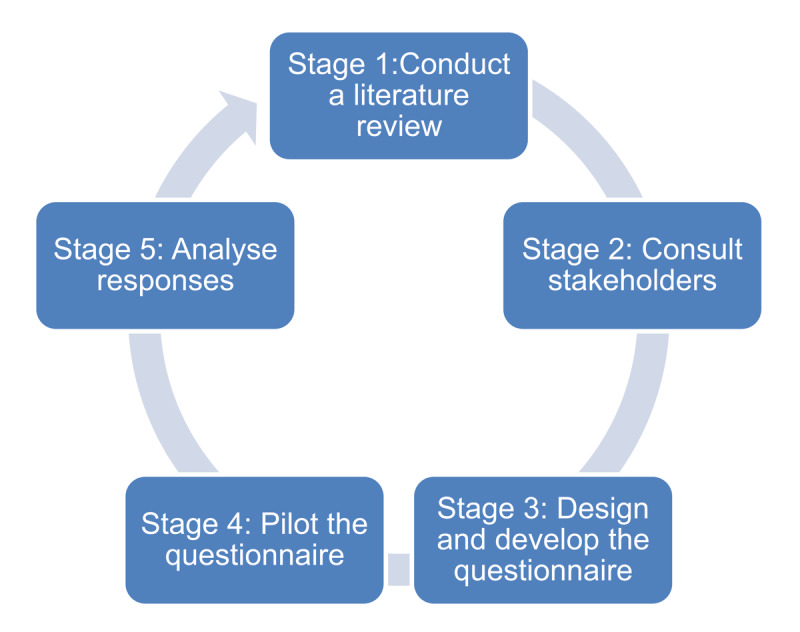
Suggested framework for qualitative questionnaire development.

The development of research data collection instruments for qualitative research is guided by two key principles: *credibility* – the effective and accurate collection of participant accounts, and *confirmability* – the accurate representation of participant responses and interpretations of the research questions, rather than those of the researcher(s) [17]. The development of a rigorously sound qualitative questionnaire follows the same principles but should also be guided by literature on questionnaire development [16]. Although the influential work of Artino et al. focuses on the design of quantitative questionnaires, a similar sequence of questionnaire design may be followed for qualitative questionnaires, albeit with some adaptations [see Figure 1].

### Stage 1. Conduct a literature review

The literature review is an important first step in developing a rigorous qualitative questionnaire. Writing questionnaire items without consulting the relevant literature can risk important themes and topics being missed and can result in poorly written questions that are difficult for respondents to interpret and answer. Consequently, researchers may end up with meaningless findings [16]. A robust literature review should therefore inform the justification of questionnaire items to ensure credibility [4].

The process will include identification of abstract and conceptual definitions of key constructs (i.e., themes, topics) and how they should be operationalised (as broad research questions and/or questionnaire items) [2]. For instance, for the qualitative researcher a seemingly simple construct such as ‘learning’ will not be defined by ‘objective’ measures such as performance on a particular exam or as an average grade performance. Driven to obtain data that are rich and layered with detail, and which represent complex and contrasting experiences and perspectives, the qualitative researcher will draw on existing literature to identify questions that generate knowledge on what learning means for participants, how, what, and why they learn. For example, Bhatt [7] asked participants to describe both their learning experiences and what they felt had been the most important consequences, and from this was able to ascertain what and how participants learned, and what outcomes had mattered to them most. For participants, learning was defined broadly, and important considerations such as motivation, career planning, and character modelling were explored.

### Stage 2. Consult stakeholders

Reflexivity in qualitative research is pivotal for ensuring credibility. It requires researchers to acknowledge their role in the research, and how their prior experiences, assumptions and beliefs are likely to influence every aspect of the research and findings [3]. Within the field of HPE research, reflexivity is particularly important since the researcher is often a clinician working in or around the field of interest [18]. By practicing reflexivity, the researcher will openly acknowledge that they are likely to be psychologically and emotionally invested in the subject matter, and in close professional proximity to research participants. By including stakeholders within research design and conduct some of the challenges associated with conducting research in such circumstances may be addressed.

‘Consulting stakeholders’ refers to a process whereby potential participants and other experts are asked for their perspective on research design, conduct, analysis and write-up. As highlighted by Gehlbach and Brinkworth [19], this process is inherently collaborative. Consultations typically begin when researchers identify (through lay and professional networks) and engage directly with stakeholders to gain expert critical feedback on the proposed research. This can be particularly valuable during the early stages of research as the researcher examines concepts, language and terminology identified in the literature and plans for later stages of the research. Here, expert stakeholders help identify what is of most relevance and value to both the aims and objectives of the research, and the population involved, and provide a broader perspective, highlighting questions and practical issues that may not otherwise have been considered (16).

Busy clinicians who conduct small scale educational research, as is the case with our students, may not have the resources or time to engage in extensive consultation with stakeholders. Nevertheless, the robustness of such small studies can be enhanced greatly by the researcher taking time to consider who their potential stakeholders might be, working within time and resource constraints to engage with at least a small number of stakeholders with a high level of interest or experience in the subject at hand (e.g., students, subject experts). The outcomes of this process (suggested questions, phrasings, and spaces for survey distribution for example) may then inform and enhance the overall research design and development of the questionnaire.

### Stage 3. Design and develop the questionnaire

All questionnaires contain two kinds of questions: those that ask for demographic and background information and those that address the research aims, objectives and research questions. Topic-based questions are mostly open and are likely to demand greater cognitive effort [20]. Literature on the writing of good interview questions as well as the questionnaire design should be consulted [2]. In the absence of interaction, writing clear and unambiguous questions is particularly important [6]. Assumptions should not be made on how participants may think, feel, or experience a particular event. In Bhatt’s study [7], participants were asked to choose an event during their undergraduate studies where a tutor demonstrated role model behaviours. Such an open question with a specific instruction enabled the gathering of diverse experiences and views. Cueing them to think of a specific event of their choice, the first question asked, ‘*describe exactly what happened including the setting, who was involved (without naming names) and what they did*’. This was then followed with ‘*what did the tutor do that made you perceive them to be a role model*’ and ‘*what impact did this have on you and/or others?*’. Questions were worded with a focus on the topic of interest (role models in undergraduate education), following a logical flow.

As a strategy for eliciting meaningful data, including closed questions can provide cues to the free-text questions [2]. For example, Emma-Okon [21] opened their questionnaire by asking participants to indicate whether they continued to teach remotely after the covid-19 pandemic, a time during which the use of remote teaching greatly increased. This closed question was then followed by a free-text question asking them to describe in as much detail as possible why they do or do not teach remotely, and if they do, what teaching methods they use. The aim was to gain insights into motivations, attitudes and challenges experienced by teachers post-pandemic that could inform the development of a hybrid teaching strategy.

To overcome barriers such as response fatigue and recruitment, questionnaires should be as short as possible [22]; between four to six questions is typical [2]. Topic questions should be clustered [6] and should include or display a reasonable amount of space for free text responses in order that participants are not overwhelmed. In keeping with qualitative ontology, participants should also be invited to share and add any other information about the topic as a final question.

### Stage 4. Pilot the questionnaire

Piloting the questionnaire with potential participants generates insight into the perspective of the people and population(s) of interest, allowing the researcher to look within their world, and be provided with expert opinion on how well the questionnaire functions, and whether it successfully addresses research aims and objectives [23]. As an example, if the topic of interest required understanding the perceptions and experiences of undergraduate students, involving a sample of students (as diverse as possible to ensure a broad range of perspectives is considered) in the development and piloting of the questionnaire will strengthen both its practical and theoretical quality. Taylor piloted the questionnaire with one Emergency Medicine colleague and two BSc students studying emergency care from two separate medical schools to assess accessibility, useability, affordance, and for proof-reading. Some clarifications were requested, and the questionnaire was amended. All three noted a particular advantage using an online questionnaire, and the ease with which participants could write about their experiences. In contrast, when piloting their questionnaire, Emma Oken et al. [21] uncovered important practical issues; their institutional Microsoft account did not permit respondents to submit the questionnaire and the form had to be transferred to another account. When analysing pilot responses, they also found participants provided very little information about their use of technology post-pandemic because they were unsure whether the questionnaire was referring to synchronous or asynchronous teaching. Consequently, the questionnaire was altered to provide possible examples of technology use alongside relevant questions (a design practice that is recommended within the literature for encouraging detailed answers [2]).

### Stage 5. Analyse responses

In qualitative research, data collection and analysis are typically concurrent and ongoing, ceasing only when thematic saturation is reached [3]. Whilst this approach has been debated more recently, reaching thematic saturation is still a common expectation among qualitative researchers [24]. This approach does not lend itself to the questionnaire method where analysis is typically conducted after data collection is complete. However, in an attempt to adhere to the concurrent and iterative data collection/analysis process, and address questions of saturation, researchers using qualitative questionnaires could choose to send invitations at multiple time points. By analyzing data as questionnaires come in, they can reflect on quality, engage in analysis, and implement any required changes prior to the next point of data collection. This would enable scrutiny of the questionnaire items, allowing the researcher to consider whether rewording is required, or whether additional questions should be included to help capture a greater depth of meaning, and align the process to a more interpretivist stance [4]. This process could also be used to consider whether sufficient thematic saturation has been reached. The practicality of this approach would of course depend on available time and resources, as well as access to participants, and may be more difficult to achieve in small-scale research conducted by clinicians for the purpose of dissertations.

## Summary

The popularity of the qualitative questionnaire amongst novice researchers is likely due to its practical appeal; questionnaires can be useful where researchers are limited in terms of funding, resources, and/or time [6]. Problems arise however, where the researcher fails to consider rigour in qualitative enquiry and proceeds under the assumption that simply asking and analysing a series of open-ended questionnaire questions constitutes qualitative research [1]. As a result, researchers may find that these open-ended questions fail to capture the voice of participants [18]. It is important to recognize that choices of methods must be rigorously justified [13]. Indeed, there are times when using the questionnaire method is not ideal. This may be the case when the sample size is likely to be small from the outset, or where the research is sensitive, and the work of building rapport with participants is thus important [13]. Misuse of the qualitative questionnaire seems to be a consequence of misunderstanding what constitutes qualitative research. Researchers must consider research rigour when developing qualitative questionnaires and the five-step framework offered here aims to support this.

## References

[1] Artino AR, Phillips AW, Utrankar A, Ta AQ, Durning SJ. “The Questions Shape the Answers”: Assessing the Quality of Published Questionnaire instruments in Health Professions Education Research. Acad Med. 2018; 93(3): 456–463. DOI: 10.1097/ACM.0000000000002002 29095172

[2] Braun V, Clarke V, Boulton E, Davey L, McEvoy C. The online questionnaire as a qualitative research tool. Int J Soc Res Methodol. 2021; 24(6): 641–654. DOI: 10.1080/13645579.2020.1805550

[3] Ng SL, Baker L, Cristancho S, Kennedy TJ, Lingard L. Qualitative Research in Medical Education: Methodologies and Methods. In: Swanwick T, Forrest K, O’Brien BC (eds.), Understanding Medical Education: Evidence, Theory, and Practice. 3rd ed; Wiley-Blackwell; 2019. pp 427–441. DOI: 10.1002/9781119373780.ch29

[4] LaDonna KA, Taylor T, Lingard L. Why open-ended questionnaire questions are unlikely to support rigorous qualitative insights. Acad Med. 2018; 93(3): 347–349. DOI: 10.1097/ACM.0000000000002088 29215376

[5] Tracy SJ. Qualitative quality: Eight “big-tent” criteria for excellent qualitative research. Qual Inq. 2010; 16(10): 837–851. DOI: 10.1177/1077800410383121

[6] Braun V, Clarke V. Successful qualitative research: a practical guide for beginners. Sage; 2013.

[7] Bhatt S. What makes for an effective role model in medical education? Examining the perceptions of undergraduate students. [master’s thesis]. Cardiff: Cardiff University; 2020.

[8] Kaiser K. Protecting respondent confidentiality in qualitative research, Qual Res Health. 2009; 19(11): 1632–1641. DOI: 10.1177/1049732309350879 PMC2805454 19843971

[9] Buckle JL. “The space between: On being an insider-outsider in qualitative research.” Int J Qual Methods. 2009; 8(1): 54–63. DOI: 10.1177/160940690900800105

[10] Karnieli-Miller O, Strier R, Pessach L. Power Relations in Qualitative Research. Qual Res Health. 2009; 19: 279. DOI: 10.1177/1049732308329306 19150890

[11] Taylor R. Exploring medical students’ perceptions of effective and ineffective clinical placements in Emergency Medicine: A Critical Incident Technique study. [master’s thesis]. Cardiff: Cardiff University; 2023.

[12] Bouchard K. Anonymity as a Double-Edge Sword: Reflecting on the Implications of Online Qualitative Research in Studying Sensitive Topics. Qual Rep. 2016; 21(1): How To Article 3, 59–67. DOI: 10.46743/2160-3715/2016.2455

[13] Bynum WE, Varpio L, Lagoo J, Teunissen PW. ‘I’m unworthy of being in this space’: The origins of shame in medical students. Med Educ. 2021; 55(2): 185–197. DOI: 10.1111/medu.14354 32790934

[14] Malterud K, Siersma VD, Guassora AD. Sample size in qualitative interview studies: guided by information power. Qual Res Health. 2016; 26(13): 1753–1760. DOI: 10.1177/1049732315617444 26613970

[15] Patrick J, Scrase J, Ahmed A, Tombs M. Effectiveness of instructor behaviours and their relationship to leadership. J Occup Organ Psychol. 2009; 82(3): 491–510. DOI: 10.1348/096317908X360693

[16] Artino AR Jr., La Rochelle JS, Dezee KJ, Gehlbach H. Developing questionnaires for educational research: AMEE Guide No. 87. Med Teach. 2014; 36(6): 463–474. DOI: 10.3109/0142159X.2014.889814 24661014 PMC4059192

[17] Tavakol M, Sandars J. Quantitative and qualitative methods in medical education research: AMEE Guide No 90: Part II. Med Teach. 2014; 36(10): 838–848. DOI: 10.3109/0142159X.2014.915297 24845954

[18] Reid A-M, Brown JM, Smith JM, Cope AC, Jamieson S. Ethical dilemmas and reflexivity in qualitative research. Perspect Med Educ. 2018; 7: 69–75. DOI: 10.1007/S40037-018-0412-2 29536374 PMC5889383

[19] Gehlbach H, Brinkworth ME. Measure Twice, Cut Down Error: A Process for Enhancing the Validity of Survey Scales. Rev Gen Psychol. 2011: 15(4): 380–387. DOI: 10.1037/a0025704

[20] O’Cathain A, Thomas KJ. “Any other comments?” Open questions on questionnaires – a bane or a bonus to research? BMC Med Res Methodol. 2004; 4(25): 2–7. DOI: 10.1186/1471-2288-4-25 15533249 PMC533875

[21] Emma-Okon B, Akomolafe R, Ayannuga O, Tombs M. Teaching pre-clinical medical students remotely in Nigeria post Covid-19 pandemic: Can past experiences shape future directions? Res Sq; 2023. DOI: 10.21203/rs.3.rs-2854973/v1 PMC11083797 38724974

[22] Edwards P, Roberts I, Clarke M, et al. Increasing response rates to postal questionnaires: systematic review. BMJ. 2002; 324(1183): 1–9.. DOI: 10.1136/bmj.324.7347.1183 12016181 PMC111107

[23] Van Teijlingen E, Hundley V. The importance of pilot studies. Nurs Stand. 2002; 16(40): 33–36. DOI: 10.7748/ns.16.40.33.s1 12216297

[24] Braun V, Clarke V. To saturate or not to saturate? Questioning data saturation as a useful concept for thematic analysis and sample-size rationales. Qual Res Sport Exerc Health. 2021; 13(2): 201–216. DOI: 10.1080/2159676X.2019.1704846

